# The Balance between Energy, Environmental Security, and Technical Performance: The Regulatory Challenge of Nanofluids

**DOI:** 10.3390/nano11081871

**Published:** 2021-07-21

**Authors:** Maria José Lourenço, João Alexandre, Charlotte Huisman, Xavier Paredes, Carlos Nieto de Castro

**Affiliations:** 1 Centro de Química Estrutural, Faculdade de Ciências, Universidade de Lisboa, Campo Grande, 1749-016 Lisbon, Portugal; xpmendez@ciencias.ulisboa.pt (X.P.); cacastro@ciencias.ulisboa.pt (C.N.d.C.); 2 IAPMEI—Agência para a Competitividade e Inovação, Direção de Proximidade Regional e Licenciamento, Campus do Lumiar, 1649-038 Lisbon, Portugal; joao.alexandre@iapmei.pt; 3 Department of Technology, University of Applied Sciences, UCLL, 3000 Leuven, Belgium; charlotte.huisman@student.ucll.be

**Keywords:** nanofluids, IoNanofluids, nanomaterials, colloids, safety, security, EU regulation, heat collector efficiency

## Abstract

Nowadays, numerous studies on nanomaterials (NMs) and Nanofluids (NFs) are account a plethora of applications. With the scientific society’s common goal of fulfilling the target of sustainable development proposed by the UN by 2030, it is necessary to combine efforts based on the scientific and technological knowledge already acquired, to apply these new systems with safety. There are thousands of publications that examine the use of NFs, their benefits and drawbacks, properties, behaviors, etc., but very little is known about the safety of some of these systems at a laboratory and industrial scale. What is the correct form of manipulating, storing, or even destroying them? What is their life cycle, and are they likely to be reused? Depending on the nanoparticles, the characteristics of the base fluid (water, propylene glycol, or even an ionic liquid) and the addition or not of additives/surfactants, the safety issue becomes complex. In this study, general data regarding the safety of NF (synthetic and natural) are discussed, for a necessary reflection leading to the elaboration of a methodology looking at the near future, intended to be sustainable at the level of existing resources, health, and environmental protection, paving the way for safer industrial and medical applications. A discussion on the efficient use of nanofluids with melanin (natural NM) and TiO_2_ in a pilot heat collector for domestic solar energy applications illustrates this methodology, showing that technical advantages can be restricted by their environment and safety/security implications.

## 1. Introduction

Currently, society faces a complex mission to fulfil in the development of energy-efficient, low-polluting (CO_2_ footprint) systems, with secondary resources and which must be based, whenever possible, in a sustainable circular economy without irreversible environmental damage.

In this context, the nanomaterials (NMs), nanofluids (NFs), and IoNanofluids (INFs) can play a key role. However, this sustainability language arrives to 2021 with little effective demonstration. If, on one hand, there is recognition of exhaustion of natural resources, as is the case of Critical Raw Materials (CRM), on the other hand there seems to be no unanimous awareness for this global problem.

The effects of the addition of NMs to base fluids and of NFs prepared by various methods are well known [1,2], and researchers recognize moderate to large changes in their thermophysical properties compared to the base fluid, namely for viscosity and thermal conductivity and, to a lesser extent, for heat capacity. Some degree of caution is also needed when navigating the literature on NFs and INFs, with results encompassing sets of totally different techniques of measurement, characterization, and preparation, without informing correctly about the errors associated to each of them (quality of the measurements and their uncertainty). There are experimental limitations, for example, in the transient hot-wire technique, not always fulfilled or accounted for [3]. This results in infinite comparisons and tables of data that are never validated [4], namely with respect to safety. There is, therefore, a need to define a special organization for the NFs, and to know the degree of toxicity appropriate to their intended use. In addition, there is an urgent need for Standard Reference Methods for NFs and for Certified Reference Materials for NMs and Standard Reference Methods and Reference Data for the NFs, only available for a particular case of silica nanoparticles dispersed in aqueous solution [5].

NFs are defined as dispersion of well characterized nanoparticles (NPs) in a fluid, usually a liquid media which, in other words, are colloids (dispersed phase of micro or nanoparticles in a given fluid, and multiphasic systems), thermodynamically unstable, but kinetically stable within a reasonable time frame. Dispersion, by definition, is a material that consists of a dispersing medium and a dispersed phase. Both the dispersing medium and the dispersed phase can be solid, liquid, or gaseous, leading to a number of possible combinations [6], see Table 1. The number of phases involved in the NF (at least two) is also important in this strategy. The biphasic system is preferable, and many times water is involved as the base fluid [7]. In the NFs case, the dispersing medium is a fluid and the dispersed phase an NM (NPs).

Although NFs are physically colloids, the upper size limit of the particles in a colloidal system varies in different definitions of the term between 100 nm [8] and 1000 nm [9]. Therefore, dispersed phases are colloids, and when we have nanoparticles/aggregates, they should be called nanofluids (or IoNanofluids, as the case may be). Rarely, the term nanocolloid is used in some literature [10], but it is not recommended as a replacement for nanofluids or IoNanofluids, to avoid confusion of those who work in the area of nanofluids, as a nanocolloid might be considered a nanomaterial [10]. Here nanocolloid is defined as “a material in particulate form, that is, it is constituted by discrete entities of compounds in amorphous or crystalline state, either organic or inorganic. The entities can also be formed by non-covalent molecular aggregates. The particles may show collective behaviour”. By distinguishing these dispersions from colloids in general, guiding our research and analysis to all systems that have particles below 100 nm (definition of NM), an organization/systemization can be achieved. The JCR report [11] and the European Initiative for Sustainable Development through Nanotechnologies (Nanofutures.eu) provides recommendations for a global, harmonized, and coherent implementation of the NM definition in any specific regulatory context at European Union and at its member states level, where safety is one main concern. The need for stabilization of NF necessarily implies the use of use of dynamic light scattering (DLS) and transmission electron microscopy (TEM) techniques or equivalent to characterize the system under study [12,13,14,15]. The stability of an NF is very important, even for safety reasons. Any sedimentation occurring and not accounted for may jeopardize the safety and operation of, for example, flow systems, clogging process equipment and causing accidents.

A discussion on the efficient use of nanofluids with melanin (natural NM) and TiO_2_ in a pilot heat collector for domestic solar energy applications illustrates this methodology.

## 2. Analysis of Existing Regulations

Nanotechnologies are increasingly used in several industrial sectors, where sectors such as aerospace, the automotive industry and transport, cosmetics, as well as energy generation and storage show the highest use [16,17,18]. Therefore, at present, nanotechnologies have widespread use and products with nanomaterials included for general consumer uses are also increasing. In fact, the use of nanomaterials in general has been consistently growing since 2016 at a compound annual growth rate (CAGR) estimated between 9% and 20%, depending on the data source [17,18,19,20,21]. The dissemination of industry sectors triggered the need to regulate the nanotechnologies market, as the specific chemical/physical behavior of the nanoforms that may drive its success as materials may also imply different properties related to health and environmental hazards when compared with the respective bulk materials.

Due to a lack of knowledge related to the safe use of nanotechnologies, some concerns have been raised about the adequacy of existing regulation in Europe, and how to address any potential regulatory gaps related to nanotechnologies [22]. The absence of regulation that addresses those concerns, is a scenario that could bias the social perception of the risk for human health, as well as for the environment. Therefore, to overcome barriers in the implementation of such a promising technology, and ensuring safety in the manufacture as well as in the identified uses, establishing regulation and enforcement is mandatory from a socio-economic point of view.

The European Union has followed an approach of adapting the existing regulatory frameworks to address the nanoforms, instead of focusing on developing new legislation from zero. This includes changes in adopted legislation on specific sectors such as cosmetics, food products and packaging, biocides, medical devices, agriculture, and electric and electronic equipment.

However, Directive 2006/42/EC, related to machinery that could have also an impact on the energy sector, does not mention nanotechnologies specifically, and in Directive 2011/65/EU, related to the restriction of the use of certain hazardous substances in electrical and electronic equipment, recital 16 is used to highlight that nanomaterials, among other potentially hazardous substances, can be included in directive’s Annex II, which is a list of restricted substances and maximum concentration values tolerated, in a complementary way to the REACH Regulation. On the other hand, the Directive 2012/19/EU, related to electrical and electronic equipment waste, foresees that the potential exposure to nanomaterials embedded may occur in the waste phase and during recycling, requiring a safety evaluation if specific treatments are needed to control this possible risk.

Considering these three directives, the impact on the nanomaterials’ uses in the energy sector came majorly from horizontal regulations on the chemicals [23] that may ban or severely restrict the use or manufacture of substances or its placing on the market. The REACH (ECHA, 2021) [24] and CLP (ECHA, 2021) [25] regulations that ensure the safe use of all substances and mixtures, alongside the specific provisions for sectors, currently form the most relevant framework of legal principles for placing nanomaterials on the market [26,27,28]. The original legal texts of the REACH and CLP Regulations, published in 2006 and 2008, respectively, implicitly apply to nanotechnologies as the included substance’s definition covers the nanoforms. However, there is no mention of nanomaterials and no definition for those forms. Additionally, as the REACH Regulation demand on the registry of substances and the requirements to the chemical safety assessment are triggered by thresholds on the annual tonnage manufactured or imported, due to low tonnages involved in nanotechnologies, those demands do not cover the nanoforms and, therefore, in the original text of the REACH Regulation there was no specific requirement to the manufacturers, importers, or downstream users to provide information on the characterization and safety of the nanoforms in the registry of substances or on chemical safety reports [22,29,30,31].

Although the risk assessment capabilities regarding nanotechnologies need to be developed to overcome current knowledge gaps [32,33], the publication in 2018 of the Regulation 2018/1881 [34] addresses explicitly the concept of nanomaterials and the concerns in REACH by changing the REACH Regulation annexes to introduce requirements specifics to the nanoforms and states that the nanomaterials risk assessment should be performed on a case-by-case basis. As the REACH Regulation addresses the burden of proof to the registrants, manufacturers, or importers, they should provide information to characterize the nanoforms and their chemical behavior, possibly meaning the need to provide scientific proofs that the nanoforms do not justify additional concerns when compared to the bulk forms. For some uses not foreseen by the registrants, this responsibility may have to be assumed by downstream users if they decided to compile their own chemical safety report.

At present, the following REACH annexes have been amended to cover the specificities related to the nanoforms:

- Annex I: General provisions for assessing substances and preparing chemical safety reports;
- Annex II: A guide to the compilation of safety data sheets [35];
- Annex III: Criteria for substances registered in quantities between 1 and 10 tonnes;
- Annex VI: Information requirements referred to in article 10 [36];
- Annex VII: Standard information requirements for substances manufactured or imported in quantities of one tonne or more;
- Annex VIII: Standard information requirements for substances manufactured or imported in quantities of 10 tonnes or more;
- Annex IX: Standard information requirements for substances manufactured or imported in quantities of 100 tonnes or more;
- Annex X: Standard information requirements for substances manufactured or imported in quantities of 1000 tonnes or more;
- Annex XI: General rules for adaptation of the standard testing regime set out in annexes VII to X;
- Annex XII: General provisions for downstream users to assess substances and prepare chemical safety reports.

In Annex I of the REACH Regulation, it is now clearly stated that the chemical safety report shall describe and cover all the forms, including nanoforms, of the substance in the scope of each registered substance. That means that when nanoforms are in the scope of registration, the included hazard assessment and risk characterization should consider the specificity of the nanoforms regarding the bulk substances.

The existence of nanoforms is currently one of the criteria for substances registered in quantities between 1 and 10 tonnes, unless those nanoforms are soluble in biological and environmental media (Annex III of REACH Regulation).

Annexes VII to X, which describe in detail which and how endpoints must be tested, as a function of the tonnage levels registered of a substance, also identify specific requirements to the nanoforms. Annex XI states that when nanoforms are covered by the registration, the foreseen several testing approaches shall be addressed separately. As the information related to those new requirements for the nanoforms may have to be included in the safety data sheets, Annex II has recently been modified accordingly.

As the legislator decided to preserve the REACH Regulation text in order to not change a Regulation that is considered complex for all the stakeholders involved, and considering that the original definition of substance included in article 3(1) covers the nanoforms, the lack of a specific definition of nanoform in the original REACH Regulation’s text was solved by the introduction in Annex VI of the overarching definition pointed out in the Commission Recommendation of 18 October 2011 on the definition of nanomaterial [37,38] that aims to provide a general basis for a common understanding in all European regulated level areas. The definition states that a nanoform means “*a form of a natural or manufactured substance containing particles, in an unbound state or as an aggregate or as an agglomerate and where, for 50% or more of the particles in the number size distribution, one or more external dimensions is in the size range 1 nm-100 nm…*”. “Particle means a minute piece of matter with defined physical boundaries; “agglomerate” means a collection of weakly bound particles or aggregates where the resulting external surface area is similar to the sum of the surface areas of the individual components and “aggregate” means a particle comprising of strongly bound or fused particles” [34].

The current Annex VI also considers that the same substance could have several nanoforms, and each one [39] should be characterized in the following terms:

- Names or other identifiers of the nanoforms or sets of similar nanoforms of the substance;
- Number based particle size distribution with the indication of the number fraction of constituent particles in the size range within 1 nm–100 nm;
- Description of surface functionalization or treatment and identification of each agent including IUPAC name and CAS or EC number;
- Shape, aspect ratio and other morphological characterization: crystallinity, information on assembly structure including, e.g., shell-like structures or hollow structures, if appropriate;
- Surface area (specific surface area by volume, specific surface area by mass, or both).

The implementation of the REACH Regulation on nanoforms relies on the capability of measuring the relevant mentioned endpoints to allow classifying a material as a nanomaterial, whether they are natural, incidental, or engineered materials and the ability to distinguish and categorize different types of nanoforms. However, there are some shortcomings in the way. Nanoscale reference materials, reliable test validation and guidance, and criteria for grouping nanoforms are issues that need to be improved upon [16,27,28,29,40].

Regarding the guidance, since 2012, the European Chemicals Agency (ECHA) has been updating the relevant guides for REACH and CLP Regulation [24,25,41]. Several chapters of the Guidance on information requirements and chemical safety assessment were updated with specific appendixes for nanomaterials, namely:

- Chapter R.6: QSARs and grouping of chemicals;
- Chapters R.7a; R.7b; R.7c: Endpoint specific guidance;
- Chapter R.8: Characterization of dose (concentration)—concentration for human health;
- Chapter R.10: Characterization of dose (concentration)—concentration for environment;
- Chapter R.14: Occupational exposure assessment.

Additionally, ECHA updated the Guidance on registration, adding an appendix specific for nanoforms, and the Guidance on the compilation of Safety Data Sheets, SDS, where the new information requirements introduced by the nanoforms are now addressed in version 4.0 published in December 2020. It should be pointed out that SDS are the more relevant information vehicle on chemicals demanded by the REACH Regulation.

The CLP’s guide, Guidance for identification and naming of substances under REACH and CLP [42], was also updated with an appendix specific for nanoforms [43]. As the nanoforms do not present new hazards, nor new exposition ways, the GHS system introduced in UE by the CLP regulation are overall still updated and less permissive to the changes challenged by the nanomaterials [44].

The guide “Guidance for Annex V”, which describes the exemptions from the obligation to register, among others, naturally occurring substances if they are not chemically modified and meet the hazard criteria stated in the Annex, was not updated with nanoforms issues. Therefore, chemically unmodified nanoforms of naturally occurring substances are treated by the REACH Regulation as any other forms of such substances. That means REACH currently takes registration of nanoforms of naturally occurring substances deemed inappropriate or unnecessary. Therefore, to cease one careful characterization of those nanoforms does not jeopardize the safety and information objectives of the Regulation.

## 3. Nanofluids and IoNanofluids

The current definition of nanomaterial only covers particulate matter materials [31], which may reveal special concerns with the risk exposition as the exposition to nanoforms is more likely for particulate materials than for matrix enclosed nanomaterials [45,46]. Therefore, for a regulation where the aim is to promote safety, the former raises more concerns [35]. Despite the narrowed scope of this definition, it is however in line with the definition of other organizations or standardization bodies such as the ISO definition of nanomaterial [7]. This being said, while the Commission definition only classifies as nanomaterials those materials aggregates or agglomerates of particles with larger external dimensions, if at least half of which have an external dimension between 1 nm and 100 nm, the ISO definition is less restrictive. Materials that have internal structures or surface structures in the nanoscale are also classified as nanomaterial, according to the ISO/TS 80004-1:2010 [7].

As any definition, the used European Commission definition on nanomaterials does not avoid borderline cases where it is not clear if the evaluation of some materials is within its scope. This is the case of nanofluids as a whole material. The statement that a nanomaterial is “a form of a natural or manufactured substance containing particles” does not clarify if colloidal nanomaterials systems, nanofluids as a whole, are a special form of a nanoform, or a mixture were the nanoparticles are dispersed in a liquid matrix. If the Commission’s definition covers only particulate materials that are solid at normal temperature and pressure [47], nanofluids may be taken as mixtures under REACH and CLP regulations, whatever the production process or the nanofluid’s nanoparticles concentration.

This seems to be the current acknowledged interpretation, but several nanomaterials are only stable in a colloidal system. As explained above, colloids are not homogeneous mixtures, they are bi- or multiphasic systems. According to the REACH Regulation, when the liquid is an essential element for the nanoparticles’ stability, it is considered as a separate substance, but for registration purposes, considering the REACH’s substance definition, the base liquid should be included in the registry as an essential additive. Additionally, the same nanoparticle, stabilized by additives in different liquid phases, should be registered separately as the additives to stabilize the substance are part of the substance. In this sense, is an NFs formulator a manufacturer or a downstream user? For clarity reasons, it might be useful, but likely difficult, to distinguish colloids intended to be used as NFs from those colloids produced only to preserve the nanoparticles’ stability, as used in some commercial nanomaterials, e.g., nano-silver and nano-silica.

However, considering the NFs as mixtures may lead to a challenge to classify those mixtures according to the CLP rules. The CLP Regulation foresees that one mixture, as is defined in it, can be classified using the relevant available information on the mixture itself or the information on the substances contained in it.

If the mixture’s classification is derived from the properties of the original pure substances that comprise it (ingredients and components), foreseeing the safe use of an NF using the individual properties of the nanoparticles, is at least questionable, as the nanoparticles interact physically with the base fluid and/or additives, profound changes deviating from the initial properties may occur, even for small NPs concentrations. Therefore, the classification of mixtures based on the available information of the constituents should be re-evaluated as a good practice for nanofluids.

However, if the classification of the mixtures is dependent on information on the mixture itself, as mentioned above, the current experimental limitations to measure some specific properties and the lack of standard reference methods to test NFs will be a shortcoming on the REACH and CLP implementation and safety goals.

In order to take full advantage of an NM, a proper understanding of its life cycle is necessary. This also applies to a NF, a proper characterization (www.nanoreg.eu, accessed on 12 January 2021) would allow all stakeholders to accurately assess its use, reuse, and recovery possibilities, as well as addressing the risks involved in its exposure to humans and the environment.

The use of NFs with natural origin NMs may solve some problems of production, scarcity, and toxicity, as is also the case of melanin and biomass [8]. Silver and copper nanoparticles are known to be strongly ecotoxic. There are also many studies on titanium dioxide and zinc oxide, showing diverging results, depending on the forms of nanoparticles studied. These studies should be considered in the work to be performed with NFs and INFs of these NMs [16,48,49,50].

INFs are complex systems, with NMs dispersed in ionic liquids (ILs). This includes molten-nanosalts. The reputation of these solvents as “environmentally friendly” chemicals is mainly based on their insignificant vapor pressure. However, the solubility of ILs in water and a number of reports documenting the toxicity of ILs for aquatic organisms highlight a real cause for concern [51]. The importance of ILs in medical and pharmaceutical applications is now relevant, and it has already been realized that its antimicrobial and cytotoxic activity may have several benefits in the future. It will then be necessary to assess the levels of toxicity and tolerances for health, avoiding uncontrolled environmental hazards [52,53]. A very complete description of synthesis and properties has been presented elsewhere, including the possible use of natural nanomaterials, from biomass, such as melanin from the cuttlefish ink sacs, and cherry stones, as sustainable and completely recyclable NMs, non-toxic for human and marine organisms [54]. The foreseen contribution of IoNanofluids for sustainable development [55], has been presented by some of the authors where the problem of the toxicity of the ionic liquids has been discussed. To better understand the ways that bacteria, plants, fish, and other maritime organisms react to the presence of ILs, and ILs + NMs, is still a challenge today, and existing results are scarce and often contradictory [56]. From the existing studies, it is clear that just for the base fluid, lipophilicity is the main driver for toxicity, and this depends very much on the cation. The anion seems to play a minor, although not negligible role, in the most common ionic liquids, where the cation belongs to the imidazolium or ammonium families.

A further sustainable idea of using nanoparticles in liquids emerged with the intent of valorizing agroindustrial and forest residues that do not have any notable value, and creating an economic relation with the food industry, giving rise to the term IoBiofluids, natural nanoparticles dispersed in an ionic liquid or other sustainable fluid. This term was firstly proposed by Carla Queirós in her MSc thesis and further developed in the PhD thesis [56,57,58], for walnut, almond, and pine nut shells, and *Hakea sericea* Schrad. fruits.

However, not many studies have been performed with IoNanofluids to date; they are mostly studied with MWCNTs and graphene, and research is absolutely indispensable to sustain any regulatory decisions on this type of nanofluids.

## 4. Nanofluid Selection Strategy

As explained in the introduction, we present here a preliminary discussion on the efficient use of nanofluids with melanin (natural NM) and TiO_2_ in domestic solar energy applications, illustrating the proposed methodology for convenient and harmless nanofluids selection. Most of this view is a revised form of the first publication on the subject, in Chapter 1 of reference [1], namely in Section 5, “A road to successful nanofluid: The nano-ecological and economic research”. There, it was said that “There has always been a key challenge in engineering to successfully apply nanomaterials into more domains as many of their physical and chemical properties are not well known, but simulations and predictions have been able to orient industry into their more promising functions, such as anticaking agent, antimicrobial protection, catalyst, coatings, cosmetic, environmental treatment, filtration, green chemistry, hardness and strength, health applications, hydrophobic treatment, lubricant, miniaturization, pigment, sorbent, sun protection. In spite of the success reached in the market with these new products, stricter regulations require manufacturers to increase the information in labels and datasheets to provide critical data about how to safely handle and use different nanomaterials or products containing nanomaterials”. These points were raised about 7 years ago, but are essentially correct today.

**Figure 1:**
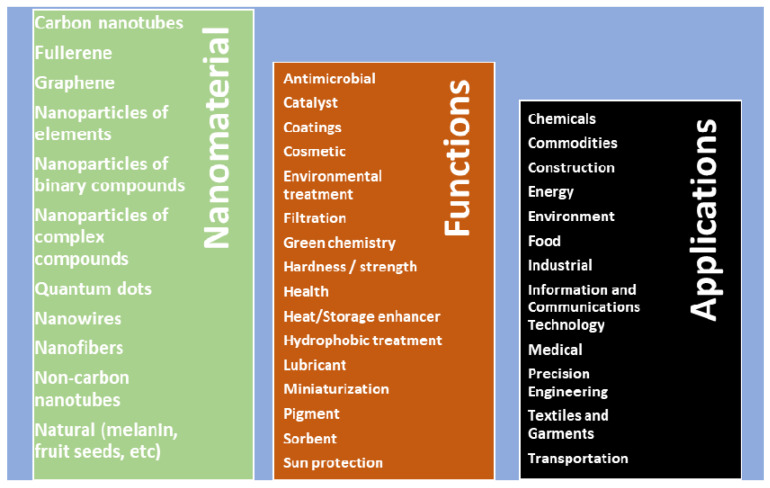
Nanomaterials, their functions and applications.

Figure 1 shows a collection of nanomaterials, their functions, and applications, adapted from [1]. It is clear from the previous analysis that the nanomaterial plays a very important role in the safety and toxicity of the nanofluids which use them. Many applications use nanofluids as a target or in the production process. A list of nanomaterial suppliers (average particle size ≤ 100 nm) is also shown in this publication.

It is necessary to define a strategy to choose a sustainable nanofluid or IoNanofluid, which includes the base liquid/fluid (BF), the NM and its characterization, the preparation and stabilization methods, the toxicity and biodegradability assays, and its recycling or waste treatment. All the definition of this strategy must be based on all the information available in the literature on the characterization and stability on NF described, to make decisions about the best NF to use for a given application. However, the results reported are frequently unreliable, contradictory, incomparable, and/or not repeatable, in particular for the scarcity of information on their preparation [59]. In this paper, the authors proposed some suggestions on the minimum amount of information that should be given on a NF to ensure comparability in results obtained for similar NFs, and to be able to reproduce the same NF in other laboratories. It will also contribute to define a reference nanofluid and round-robin measurements performed with state-of-art or certified accurate instruments, to permit the assessment by each laboratory of the quality of their instrumentation, in a fashion similar to that which we have recently proposed for the IUPAC reference ionic liquid, 1-hexyl-3-methylimidazolium bis(trifluoromethyl)sulfonyl imide, [C6mim] [(CF3SO2)2N] [60]. This has been one of the objectives of the recent COST Action Nanouptake [61] and its ongoing COST Innovation Grant [62].

**Figure 2:**
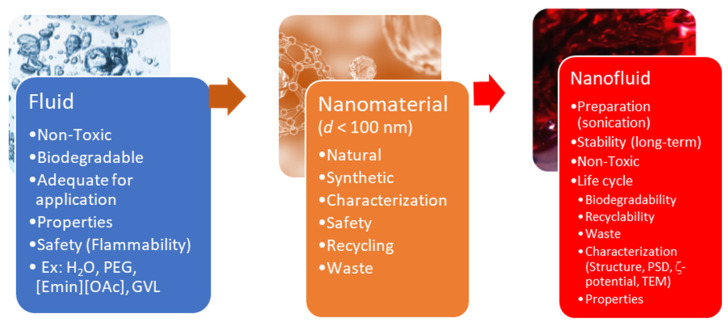
Elucidative diagram to orient researchers/users to decide about the best methodology to prepare a sustainable NF/INF, adhering to safety, health, and environmental regulations.

Figure 2 tries to devise a scheme to respond the question of choosing a sustainable NF.

The importance of the existing regulations, both for safety and environmental concern, decides if any selected fluid and nanomaterial combination is adequate for a given application of the projected NF. The choice of the NM, as already discussed before [59] is extremely important, namely, if it is acquired and not synthesized, the choice of the manufacturer and the guarantee that the sample has the specifications claimed.

Ideally, NMs of natural origin should be used in the long term, such as melanin and others, to be determined/unearthed in the future. However, their availability is still a problem, so other synthetic materials have to be used for the next years. In addition, in the NF production, extreme care must also be taken, if long alkyl chains surfactants are used for dispersion stabilization, as they cannot pass the environmental scrutiny.

A final comment on characterization, namely on the measurement of properties of the nanofluids. As they are at least biphasic systems, care has to be taken if NPs concentrate, by adhesion forces, on the surfaces of the measuring sensors, providing additional heat or mass transfer mechanisms which would mask the experimental values obtained. This is the case, for example, for thermal conductivity, one of the most important properties for conductive and convective heat transfer applications. As explained by Nieto de Castro and Lourenço [3], who critically reviewed the best available techniques for the measurement of thermal conductivity of fluids, with special emphasis on transient methods and their application to ILs, NFs, and molten salts, several commercial instruments do not guarantee a correct use of the physical models used for the measurement. The transient hot-wire method, being the recommended method of measurement, has to fulfil several necessary conditions to secure accurate measurement of the effective thermal conductivity of two-phase systems comprising nanoscale particles of one material suspended in a fluid phase of a different material, our NFs [63]. Tersinidou et al. [64] have reported new data for ethylene glycol with added CuO, TiO_2_, and Al_2_O_3_ NPs and for water with TiO_2_, and Al_2_O_3_ or MWCNTs, using both the transient hot-wire and hot-disk instruments, compared the results obtained with the two instruments and with other available data. They then demonstrated the importance of quality measurements for heat transfer studies, emphasizing the scatter of data obtained in different laboratories, also without proper characterization of the NMs used.

## 5. Heat Transfer Pilot Study

One of the many uses for heat transfer fluids is in flat-plate solar collectors, widely used for water heating in residential buildings. An active system uses a pump to circulate the heating fluid, while a passive one uses the thermosiphon principle, with the liquid being transported by natural convection. Solar water heating systems can be further subdivided in direct systems, where the fluid being circulated is the water to be heated, and indirect systems, where the water is heated through a heat exchanger and the fluid being circulated is not physically in contact with the water. The authors will not dwell on the advantages and disadvantages posed by any of these systems. For our study, we have used a simple home-made flat-plate collector with a pump (active) and a heat exchanger (indirect) in order to test the performance changes that arise from the introduction of melanin NPs and TiO_2_ in different base fluids. As baseline for the comparison, measurements with ultrapure type 1 water, commercially available propylene glycol mixture with water (30/70 *w*/*w*)(A2BRIOS, Solux Fripol 30 Plus, used as a heat transfer fluid in thermal solar applications, antifreeze, m.p. −14 °C, biodegradable, Regulation (CE) Nº 648/2004 Annex II and III), and a mixture of water (7/93 *w*/*w*) with the ionic liquid 1-ethyl-3-methylimidazolium methanesulfonate, [C_2_mim][CH_3_SO_3_] (ST35, proposed as an heat transfer fluid, m.p. 34.8 °C) [65].

Melanin can be obtained from several sources, such as ink sacs of squids and octopuses, sponges, fungi, banana peels, tea leaves, and different types of beans, allowing waste reuse and recycling. In our laboratory, melanin was extracted from cuttlefish, chemically and thermally described in reference [66], and has a *C*_P_ of approximately 2 J·g^−1^·K^−1^ at room temperature and 6 J·g^−1^·K^−1^ at 100 °C (same order of magnitude as water). The nanofluid was prepared with a 0.02% *w*/*w* concentration of melanin NP’s in water and in the solar fluid (corresponds to a volume fraction of 0.0001). Natural melanin is characterized by the presence of well-organized, non-porous, and spherically shaped granules, with diameters smaller than 120 nm, originating a more homogeneous morphology, typical of an NM [66].

**Figure 3:**
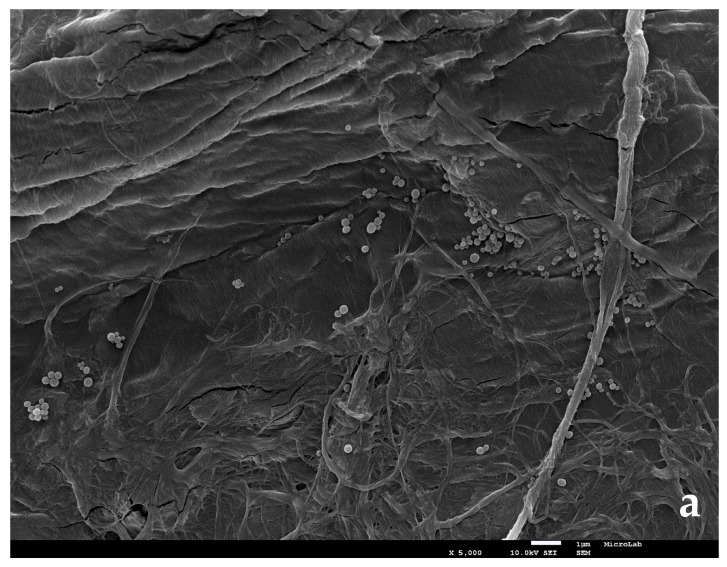

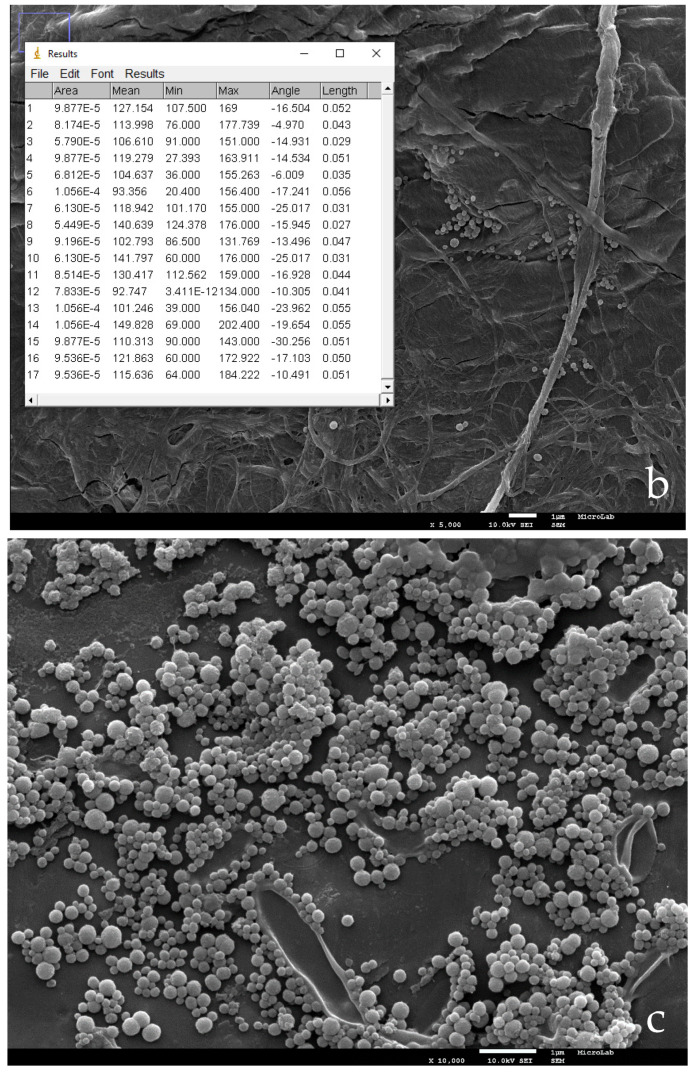

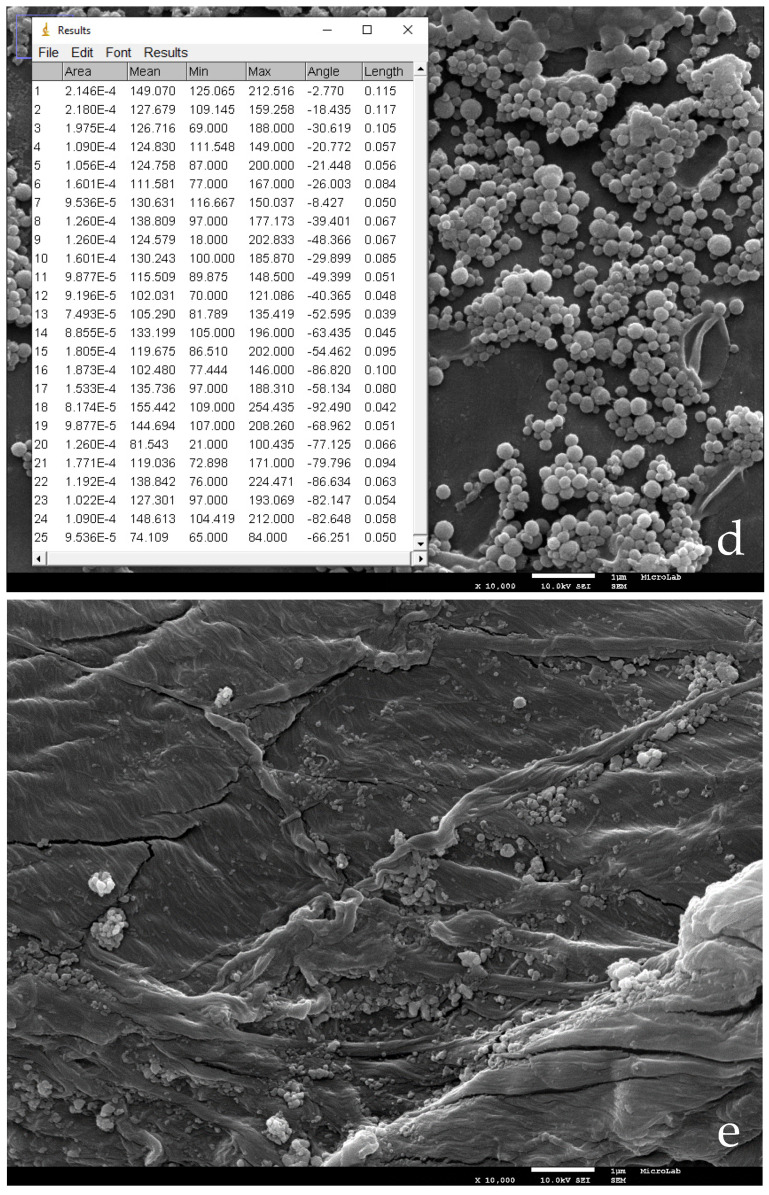

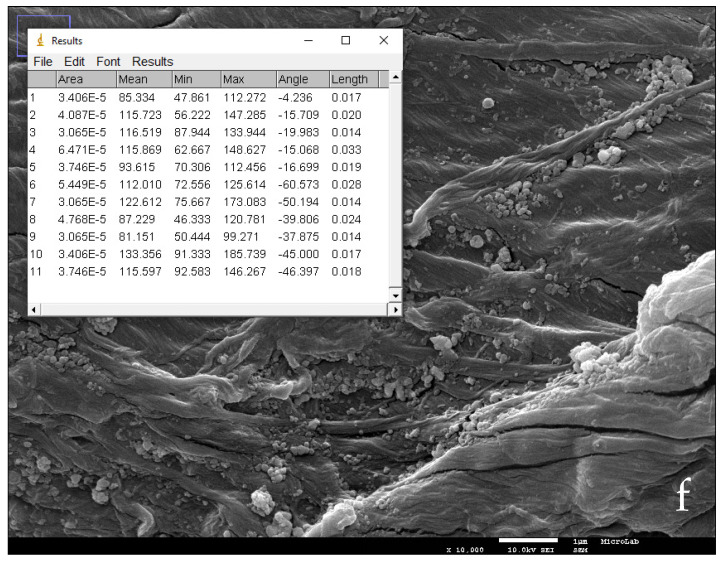
SEM images of melanin and TiO_2_ nanoparticles used in this work. (**a**–**d**), for melanin; (**e**,**f**) for TiO_2_. Explanation in the text. Measurement bars in microns. Images magnification: (**a**,**b**)—×5000, (**c**) to (**f**)—×10,000.

Images were obtained in a FEG-SEM microscope from JEOL (model JSM 7001-F), in MicroLab-Electron Microscopy Laboratory at IST, ULisboa. Figure 3a shows the image of melanin NPs of the water nanofluid, in filter paper, after water evaporation in air, and Figure 3b shows the NPs size determination, using ImageJ software (https://imagej.nih.gov/ij (accessed on 23 March 2021)) found to have an average of 44 nm for 25 measurements. Figure 3c shows an image obtained by letting one drop of the NF dry in the chamber, and Figure 3d shows the particles diameter determination found to have an average value of 70 nm.

Titanium oxide was used in the preparation of the TiO2 NFs (rutile, 10–30 nm, 99.9%, from IoLiTec; NO-0046-HP; LOT: HNO046007). Its analysis was determined by EDS, and confirmed the manufacturer’s purity. Figure 3e shows the image of TiO_2_ NPs in filter paper and after water evaporation in air, and Figure 3f shows to have an average of 20 nm for 11 determinations, also confirming the manufacturer’s characteristics. The particles show some aggregated groups.

The NFs were prepared by a step 2 preparation method [1], by adding the nanoparticles of the natural melanin using a sonicator (Hielscher, UP200HT), with a probe S26d40, optimized for an amplitude of 70%, a pulse cycle of 70% (max. 11 W) and a frequency of 25 ± 1 Hz for 2 + 1.5 min.

**Figure 4:**
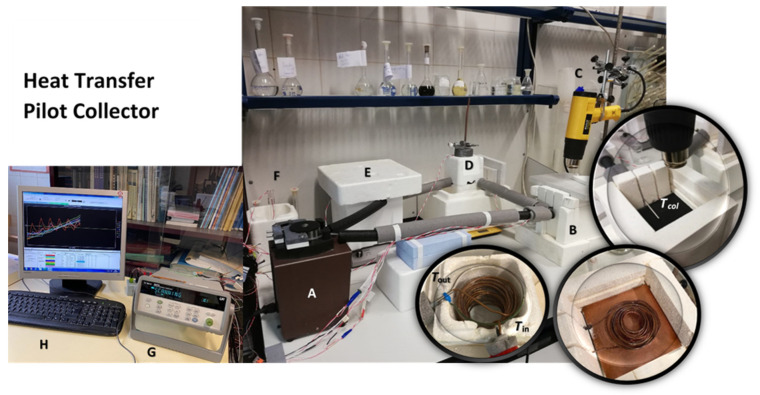
The heat transfer pilot collector. A—Peristaltic pump; B—Absorbing panel, with selective absorptive paint. *T*_col_ is the surface thermometer of the panel; the copper serpentine is for fluid circulation; C—Hot air gun; D—Heat storage (water), showing copper serpentine. *T*_in_ and *T*_out_ are the measuring thermometers; E—Fluid reservoir; F—Room temperature measurement; G—Multiplexer; and H—Monitor with heating curves.

The heat transfer collector model is made of insulating bricks JM 23 (Morgan Thermal Ceramics), for the casing, copper capillary tubing (i.d. 0.9 mm) rolled up in a double cone shape, and sandwiched between two 0.1 m by 0.1 m plates, B. The absorber plate can be made of aluminum, copper, or copper treated with a black photosensitive pigment developed and patented [67,68], containing also melanin of natural origin from cuttlefish. It is completely naturally degradable and has good thermal properties, such as heat capacity per unit volume, 6500 kJ·m^−3^·K^−1^ [66]. Figure 4 shows the layout of the heat collector pilot system.

A peristaltic pump takes care of circulating the fluid in the system, from the heat collector B to the water reservoir D, a simple glass recipient inside insulating foam where more rolled up copper tube is used to facilitate the heat exchange. For short term tests, namely in winter time, the solar radiation can be replaced by a hot air gun.

The temperatures of the flowing stream were measured by placing 10 Pt-100 resistance thermometers, calibrated between 20 and 80 °C, to within 0.08 K connected to a multiplexer (Agilent LXI Data Acquisition/Switch Unit, Model 34972A), to measure the ambient temperature, collector absorber plate temperature, the storage reservoir temperature, as well the temperatures of the stream at the entrance and exit of the absorbing unit and the storage reservoir, under every ten seconds. Mass flow rate was measured by weighing, in a given time interval, the change in mass of the stream for a given time interval.

**Figure 5:**
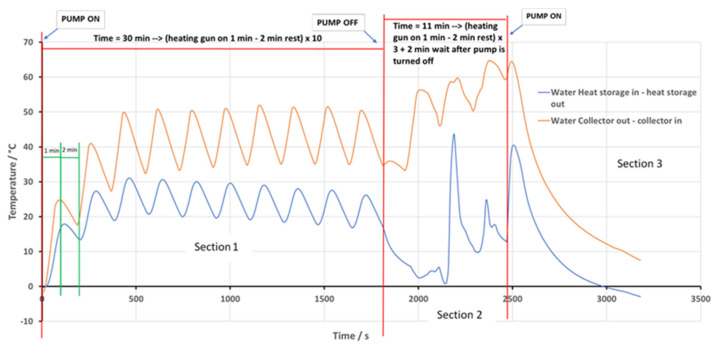
The temperature profiles in the storage reservoir (in blue), and in the heat collector (in orange), as a function of time, expressed as *T*_out_ − *T*_in_, in K, for a run with water.

The methodology used follows that reported previously [69] for graphene nanofluids, and a preliminary phase of this study was presented elsewhere [70]. The circuit itself is first filled in with the heat transfer fluid. Afterwards the system goes through a cycle where, first, the fluid under the absorber plate heats up for 1 m, followed up by a period where it is circulated so that the heat is transferred to the water storage reservoir. This cycle takes place 10 times (Section 1). After that, the pump is stopped, to observe the temperature evolution in static mode (Section 2), using three cycles (1 m heat + 2 m stop). The pump is switched again and the cooling profile is observed (Section 3). For every heating fluid the complete test is repeated three times. The heating scheme is shown in Figure 5.

The efficiency of the heat collector, defined as the ratio of the usable thermal energy to the incident hot air gun (solar radiation, when applicable) can be calculated using Equation (1):

(1) $$\eta =\frac{\dot{m}C_{p}}{AI}\left(T_{out}-T_{in}\right)$$

where $\dot{m}$ is the mass flow rate, $C_{p}$ the heat capacity of the fluid/nanofluid, *A* the collector area, *I* is the intensity of the hot air heat source, the heat gun power per unit area of the collector (≈30 kW/m^2^), and $T_{out}\;\mathrm{and}\;T_{in}$ are the average temperatures of the *out* and *in* flow streams in the collector or water reservoir, respectively.

The heat capacity and the density of the nanofluid, $\rho_{nf\;},\;C_{p_{nf}}$, can be calculated from the proposed mixing rules given by Equations (2) and (3):

(2) $$\rho_{nf}=\phi_{np}\rho_{np}+\left(1-\phi_{np}\right)\rho_{bf}$$

(3) $$C_{p_{nf}}=\phi_{np}C_{p_{np}}+\left(1-\phi_{np}\right)C_{p_{bf}}$$

where $\phi_{np}$ is the volume fraction of the nanoparticles in the nanofluid, $\rho_{np}\;\mathrm{and}$ $C_{p_{np}}$ are the density and the heat capacity of the nanoparticles, respectively, and $\rho_{bf}\;\mathrm{and}$ $C_{p_{bf}}$ the density and heat capacity of the base fluid, respectively. The volume fraction of the NPs can be calculated from the respective mass fraction, $\omega_{np}$, using Equation (4):

(4) $$\phi_{np}=\frac{1}{1+\left(\frac{1-\omega_{np}}{\omega_{np}}\right)\left(\frac{\;\rho_{np}}{\rho_{bf}}\right)}$$

Values for the density and heat capacity of the base fluids were taken from selected sources [71,72] and averaged within the temperature interval of the flowing streams, except for ST35 + water (7/93 *w*/*w*), which was obtained for mass combination rule from pure densities and heat capacities of water and ST35 [66]. Density of melanin nanoparticles was taken from reference [66], 1620 kg·m^−3^, and an average heat capacity value of 4000 J·kg^−1^·K^−1^ was used. For TiO_2_, a bulk density of 310 kg·m^−3^ and an average heat capacity of 805 J·kg^−1^·K^−1^ was used [73].

If the same mass flow rate is used, we can calculate the efficiency of the pilot heat collector with respect to that of water, under the same heat flux applied (or solar irradiance):

(5) $$\frac{\eta_{HTF}}{\eta_{Water}}=\frac{C_{p_{HTF}}}{C_{p_{Water}}}\frac{{\left(T_{out}-T_{in}\right)}_{HTF}}{{\left(T_{out}-T_{in}\right)}_{Water}}$$

The value of $\left(T_{in}-T_{out}\right)$ was calculated from the experimental data (see Figure 5), using the average of all data points. The relative efficiency of the heat collector, for the different HTFs used, can be observed in Table 2.

The efficiency of the heat collector was found to increase 9% with water + melanin NF, 11% with the solar fluid + melanin, 10% with the ST35 + water (7/93) + melanin, and 4% with the TiO_2_ nanofluid, showing that melanin is a true heat transfer enhancer with either water, the solar fluid, or the ionic liquid + water system. It is interesting that the solar fluid alone seems to be 9% more efficient than water, an unexpected result, probably due to the data for heat capacity, only found for 25 °C. The mixture ST35 + water (7/93) is, in this range of temperatures, 8% less efficient of water, but the addition of melanin recovers this deficiency completely, the efficiency becoming almost identical, a factor that will be better discussed in a new publication of this group in the thermophysical properties of ST35 + water mixtures and their IoNanofluids with melanin.

**Figure 6:**
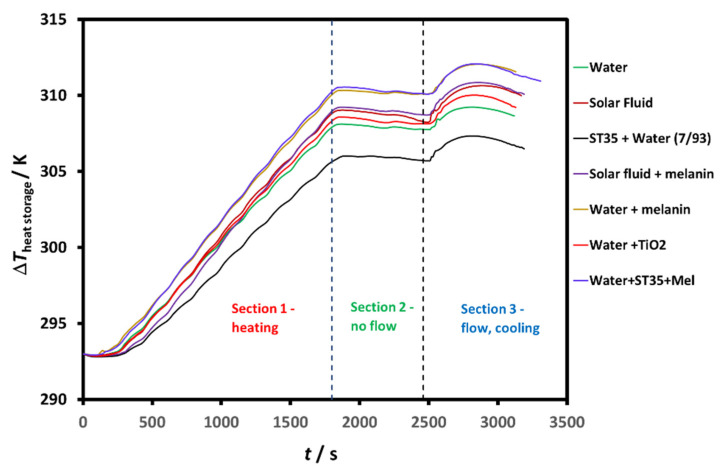
Example of temperature increase in the water reservoir (heat storage) for the different heat transfer fluids used in this study. $∆T_{\mathrm{heat}\;\mathrm{storage}}=T_{max}-T_{min}$.

Another way of visualizing this effect is by measuring the temperature change of the water reservoir. This is the most important parameter to analyse the performance of the fluid, namely the difference in the out temperature and the highest temperature achieved in the water reservoir. In this way, we can see how much heat has been transferred to the water (including heat losses in the pipes), and how the temperature does not decay much after switching off the pump. An example of the temperature variation in the water reservoir is shown in Figure 6, where equivalent results using all the fluid displayed in Table 2 are compared. The effect of melanin in the ST35 + water mixture is remarkable (27% increase in efficiency and 4.62 K).

If we calculate the efficiency using $∆T_{\mathrm{heat}\;\mathrm{storage}}$, we can see that the overall behaviour of the HTFs changes slightly, probably due to the different heat losses in the piping, inside the reservoir. The value of this temperature increase in the storage reservoir was obtained by selecting the maximum and the minimum temperatures of the reservoir during the total cycle. Table 2 also displays these values and the efficiency ratios. It can be seen that here the efficiency of the water + melanin system increased 17%, the ST35 + water (7/93) + melanin increased 12%, while for the other HTFs the variation is similar, although only increases against water for the TiO_2_ nanofluid.

The strategy for choosing a sustainable nanofluid can now be applied. For technical reasons, we have four candidates, as all the four nanofluids studied increase the efficiency of the base fluids, water + melanin nanofluid being the best. For environmental reasons, as melanin is a non-toxic, natural nanomaterial, the three nanofluids can be recommended, as they can also be recycled or disposed. The solar fluid + melanin nanofluid has only a very small increase in the efficiency when melanin is added. The stability of the melanin nanofluid systems, especially in water, was the object of publications [68,69]. It can be said the dispersions are extremely stable, lasting without significant sedimentation for more than one year.

With respect to TiO_2_, by far the most studied type of nanofluids, with several base fluids [1,2,4,64], a complete study (UV-VIS absorbance as a function of time) not described here, and the subject of a future publication, showed that the nanofluid with the TiO_2_ used in this study is stable for more than three weeks. Titanium dioxide does not pose additional environmental problems to those already existing with its application in toothpaste, food colorants, and nutritional supplements on a large scale, as well as sunscreens and cosmetics [74]. However, human exposure may occur through ingestion and dermal penetration, or through the inhalation route, during both the manufacturing process and use [48,74], which cause potential human health risks, mining the safety of its use. On the contrary, they may prevent cancer, protecting the skin against the adverse effects of UV radiation [75]. Therefore, we can conclude that there is a need to resolve these controversies by making further investigations on the safety issues of TiO_2_ nanoparticles (human exposure, animal studies, etc.), their harmless dimensions, and their concentrations in the fluids/pastes used.

Therefore, the use of TiO_2_ nanoparticles is not recommend, as a cautionary attitude. We are then left with the melanin nanofluids, for environmental, safety, and technical issues.

Finally, a short note must be added about recycling of nanofluids. Aqueous systems of TiO_2_ and melanin can be separated by evaporation, even by sun exposure (in Portugal). Melanin can also be mechanically filtered, as it has spherical particles, with a mesh less than 50 nm. TiO_2_ can also be recycled by precipitation (eventually using chemical treatments, pH modification, or others) and subsequent centrifugation. No methodologies are yet available for large scale production of nanofluids, and this is an urgent need for future research. For the case of the solar fluid, melanin can be separated in the same way as the water systems, while for the ST35 + water melanin nanofluid, melanin can be mechanically filtered, similar to water, as the viscosity of the base fluid is not much higher than that of water.

## 6. Conclusions

The advent of relevant challenges for sustainable societies, in fields as diverse as health, food security, or energy, requires new approaches for the operation of the systems involved, where it is necessary to consider the permissible levels of any substance in the environment without disturbing its balances, evaluating the toxicity, biodegradability, bioaccumulation and possible health effects. Human food and transport systems in the future (8.5 billion persons by 2030) will have to consider the release of generated CO_2_, with the direct consequences this implies.

The implementation of a regulatory framework that can be a driver of nanotechnologies development is essential to its acceptance by society. However, there is still room for improvements to generate more knowledge, which in turn will avoid regulations issues becoming barriers with unnecessary demands, instead of drivers to future development, being more effective in environmental and health preservation. There is a need for clarification on how to include nanofluids (and colloids) in the reglementary framework, to avoid their classification as nanomaterials, as they are in fact multiphasic systems, and not mixtures, in the thermodynamics framework.

A strategy to select a given nanofluid or IoNanofluid for a given application, bound by the environmental and safety regulatory frameworks is proposed, and illustrated be their use in a heat (solar) collector, specially built for studying the relative efficiency compared to water or solar fluid. It is shown that melanin is a candidate with high potential to be used in nanofluids of these solvents, while TiO_2_, although technically feasible, suffers from several environmental and safety concerns, still far from accessing its utilization in nanofluids.

NFs have the potential to play an important role in increasing the sustainability of a wide range of sectors. NMs, NFs, and INFs could contribute to more sustainable uses through cleaner, less wasteful production processes and can substitute conventional materials, leading to savings in raw materials and energy. However, it is necessary to define a reference nanofluid, very well characterized, metrologically, morphologically, structurally, and property wise, to decrease the current spread of literature data, paving the way for better measurements and data quality in the nanofluids field.

## Figures and Tables

**Table 1:** Different types of colloids.

Dispersed Phase	Dispersed Medium	Type of Colloid	Examples
Solid	Solid	Solid Sol	Glass, Coloured Glasses, and Gems
Solid	Liquid	Sol	Paints, Cell fluids, Blood, Mud, Ink, Nanofluids, IoNanofluids, and IoBiofluids
Solid	Gas	Aerosol	Smoke, Smog, Dust, and Volcanic ash
Liquid	Solid	Gel	Cheese, Butter, Jelly, Gelatine, Toothpaste, Natural rubber
Liquid	Liquid	Emulsion	Milk, Hair cream, Mayonnaise, and Brewed coffee
Liquid	Gas	Aerosol	Fog, Mist, Cloud, Hair sprays, and Parfum
Gas	Solid	Solid Sol	Pumice stone, Foam rubber (sponge)
Gas	Liquid	Foam	Froth, Whipped cream, Soap lather, and Fire retardant

Note: The dispersed phase can have the aspect of solid particles, liquid, or gas bubbles.

**Table 2:** Relative efficiency of the pilot heat collector for different heat transfer fluids and nanofluids.

HTF	Water	Solar Fluid ^a^	ST35 + H_2_O (7/93)	H_2_O + TiO_2_ Nanofluid ^b^	H_2_O + Melanin Nanofluid ^c^	Solar Fluid + Melanin Nanofluid ^c^	ST35 + H_2_O (7/93) + Melanin Nanofluid ^c^
*ρ*/kg·m^−3^	980.8	996.2	999.3	1007	980.9	1000	999.4
*C*_p/_J·kg^−1^·K^−1^	4183	3915 ^d^	4004	4176	4183	3915	4004
$T_{out}-T_{in}$/K	23.57	27.40	22.64	24.53	25.78	27.99	27.03
*η*_HTF_/*η*_Water_	1.00	1.09	0.92	1.04	1.09	1.11	1.10
$∆T_{\mathrm{heat}\;\mathrm{storage}}$	16.35	17.74	14.53	17.16	19.12	18.02	19.15
*η*_HTF_/*η*_Water_	1.00	1.02	0.85	1.05	1.17	1.03	1.12

^a^ PEG + water mixture (30/70, *w*/*w*); ^b^ TiO_2_ mass fraction *w*_np_ = 0.0006, volume fraction *ϕ*_np_ = 0.0021; ^c^ Melanin mass fraction *w*_np_ = 0.0002, volume fraction *ϕ*_np_ = 0.0001; ^d^ This value was assumed at 25 °C, as no data was found for this mixture at higher temperatures.

## Data Availability

Data used in this paper, other than experimentally obtained, have been published elsewhere, the corresponding citations being in the text, as references.
